# Interplay of Static and Dynamic Disorder in the Mixed-Metal Chalcohalide Sn_2_SbS_2_I_3_

**DOI:** 10.1021/jacs.2c13336

**Published:** 2023-05-30

**Authors:** Adair Nicolson, Joachim Breternitz, Seán R. Kavanagh, Yvonne Tomm, Kazuki Morita, Alexander G. Squires, Michael Tovar, Aron Walsh, Susan Schorr, David O. Scanlon

**Affiliations:** † Thomas Young Centre and Department of Chemistry, University College London, 20 Gordon Street, London WC1H 0AJ, U.K.; ‡ Helmholtz-Zentrum Berlin für Materialien und Energie, Structure and Dynamics of Energy Materials, Hahn-Meitner Platz 1, 14109 Berlin, Germany; ¶ Thomas Young Centre and Department of Materials, Imperial College London, Exhibition Road, London SW7 2AZ, U.K.; § Department of Geosciences, Freie Universität Berlin, Malteserstraße 74-100, 12249 Berlin, Germany; ∥ Department of Chemistry, University of Pennsylvania, 231 S. 34 Street, Philadelphia, Pennsylvania 19104-6323, United States

## Abstract

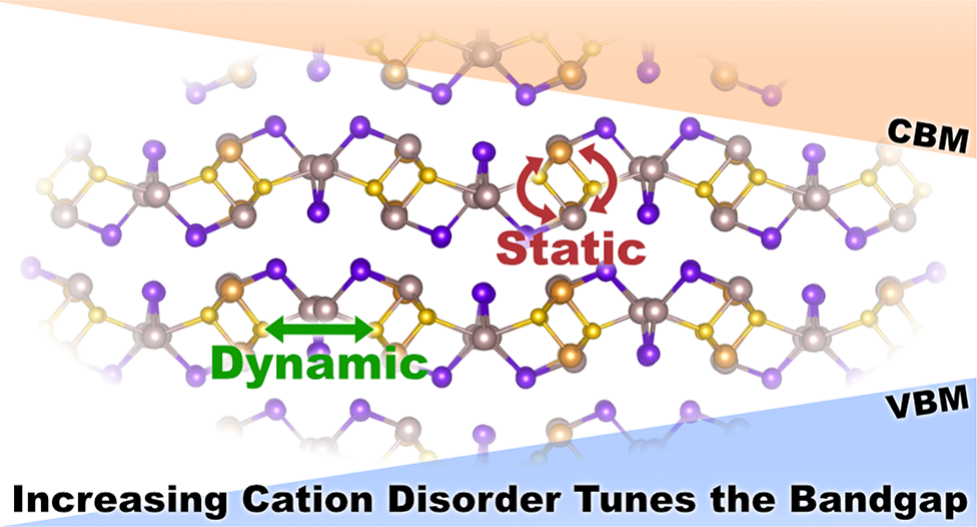

Chalcohalide mixed-anion crystals have seen a rise in interest as “perovskite-inspired materials” with the goal of combining the ambient stability of metal chalcogenides with the exceptional optoelectronic performance of metal halides. Sn_2_SbS_2_I_3_ is a promising candidate, having achieved a photovoltaic power conversion efficiency above 4%. However, there is uncertainty over the crystal structure and physical properties of this crystal family. Using a first-principles cluster expansion approach, we predict a disordered room-temperature structure, comprising both static and dynamic cation disorder on different crystallographic sites. These predictions are confirmed using single-crystal X-ray diffraction. Disorder leads to a lowering of the bandgap from 1.8 eV at low temperature to 1.5 eV at the experimental annealing temperature of 573 K. Cation disorder tailoring the bandgap allows for targeted application or for the use in a graded solar cell, which when combined with material properties associated with defect and disorder tolerance, encourages further investigation into the group IV/V chalcohalide family for optoelectronic applications.

## Introduction

Lead-halide perovskites have been at the forefront of photovoltaic (PV) research in the past decade due to their rapidly increasing efficiencies.^1,2^ Much of their excellent optoelectronic performance has been associated with the *n*s^2^ electronic configuration of the lead cation in combination with the halide anion, leading to materials that are highly defect tolerant.^3^ This characteristic feature can guide the search for “perovskite-inspired materials” which reproduce the optoelectronic performance while avoiding the use of toxic elements.^3−5^ On the other hand, metal chalcogenides have not managed to achieve the same efficiencies as halide perovskites, predominantly due to intrinsic defects limiting their performance.^6−9^ They have, however, shown improved water and air stability, compared to their halide counterparts, which is essential in the development of PV modules that can compete with the device lifespans of silicon-based technologies.^10^ As a result, metal chalcohalides offer a promising alternative to both classes of materials, combining the ambient stability of metal chalcogenides with the desirable cation–anion bonding of halide perovskites. Initial solar cells containing these materials have shown promise, achieving power-conversion efficiencies (PCEs) > 4%.^11−14^

In this work we focus on Sn_2_SbS_2_I_3_, a promising mixed-metal chalcohalide from the A^II^_2_B^III^Ch_2_X_3_ class of materials consisting of *n*s^2^ lone pair cations from groups 14 (A = Sn, Pb, Ge) and 15 (B = Sb, Bi). The first device was fabricated by Nie et al.^11^ and obtained a PCE of 4.04% using a single-step, solution-based chemical deposition process. Although this material family has been known since the 1980s,^15−18^ it has seen renewed interest in recent years, primarily due to its potential for optoelectronic applications.^5,11,19−21^

**Figure 1:**
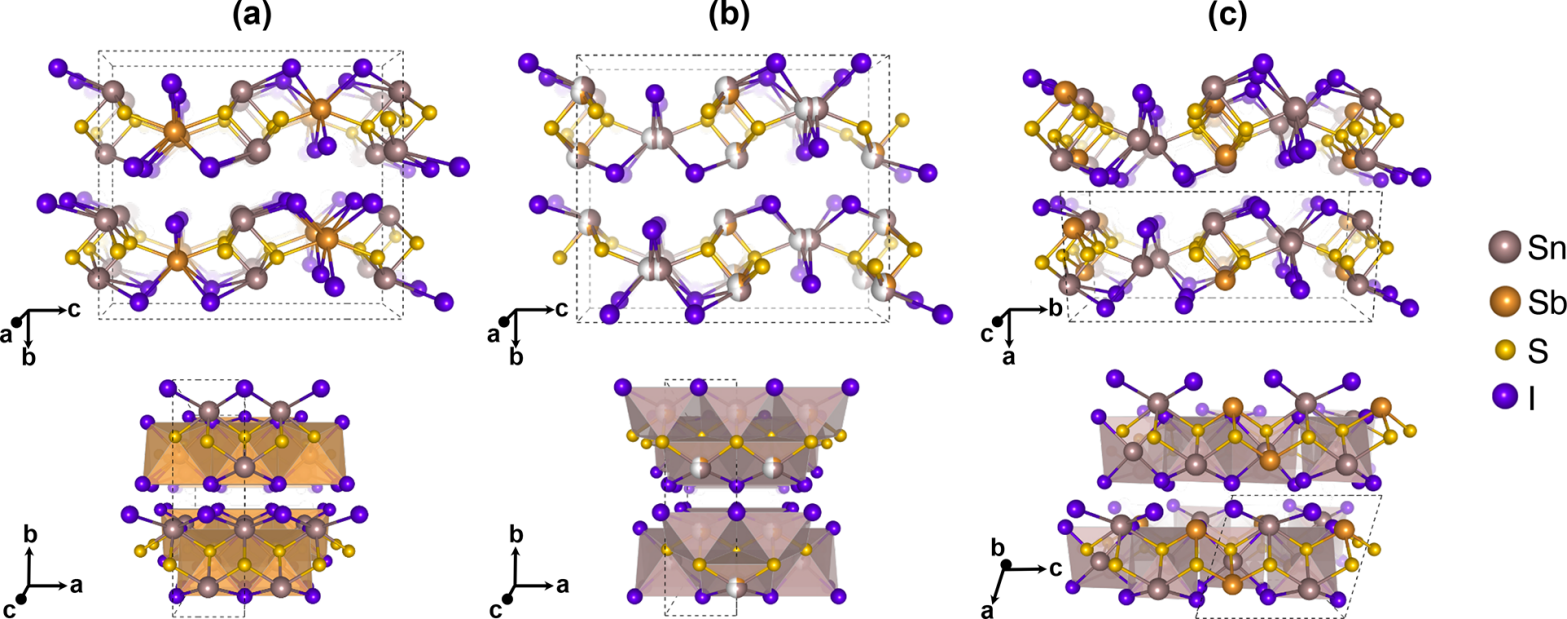
(a) The experimentally determined conventional unit cell for Sn_2_SbS_2_I_3_ from Olivier-Fourcade et al.,^15^ (b) our experimentally determined unit cell for the room-temperature structure with Sn and Sb switched, and (c) the theoretically determined *P*2_1_/*c* ground-state structure. The atom colors are Sn = beige, Sb = orange, S = yellow, and I = purple, and sites with 50% occupancy are indicated by white hemispheres. All figures were generated using VESTA.^26^

Sn_2_SbS_2_I_3_ was first synthesized by Olivier-Fourcade et al.^15^ and characterized using X-ray diffraction (XRD). They determined that Sn_2_SbS_2_I_3_ crystallizes into the *Cmcm* centrosymmetric space group and that the structure is made up of infinite Sn_2_S_2_ chains directed along the *a* lattice vector connected by distorted trigonal prismatic SbI_6_ polyhedra along *c*, as shown in Figure 1(a). The MX_6_ polyhedra are trigonal face sharing and bicapped by the chalcogen atoms from the neighboring M_2_Ch_2_ chains. Ibanez et al.^16^ also assigned the *Cmcm* space group, but report a reduction in the *R*-factor from 0.105 to 0.066 when Sb is shifted from the 4c Wyckoff site to the 8f site (a displacement of the Sb atom along the *c* lattice vector) with a 50% partial occupancy, Figure S2(a). A recent computational study into Sn_2_SbS_2_I_3_ proposed that the experimentally observed *Cmcm* structures were an averaging of multiple *Cmc*2_1_ configurations, with the Sb site corresponding to the 8f Wyckoff site in the *Cmcm* configuration.^22^ This non-centrosymmetric *Cmc*2_1_ structure gives rise to the prediction of spontaneous electric polarization in the material family, which could enhance charge carrier separation and enable open-circuit voltages above the bandgap to be achieved.^23^

Dolgikh^17^ also synthesized Pb_2_SbS_2_I_3_ and determined it to be isotypic to Sn_2_SbS_2_I_3_. A different disordered structure for Pb_2_SbS_2_I_3_ was determined by Doussier et al.^24^ in which the cation sites along the Pb_2_S_2_ chains are replaced with a mixed Pb_0.5_Sb_0.5_ occupancy (Pb(chain)/Sb(chain) site) and where Pb, rather than Sb, occupies the MX_6_ metal-halide polyhedra site, Figure S2(c). This second Pb site, Pb(polyhedra), is split between two equivalent 8f subpositions close to the 4c Wyckoff site. They argue that this configuration of cations—with the divalent A^II^ cation occupying the MX_6_ polyhedron and a 50/50 occupancy of A^II^/B^III^ cations along the M_2_Ch_2_ chains—would also be found in Sn_2_SbS_2_I_3_ and that the true structure of this material had been missed due to the similar X-ray scattering factors of Sn and Sb.^25^ The disordered mixed-cation sulfur chains have also been observed in Pb_2_BiS_2_I_3_ and Sn_2_BiS_2_I_3_, although no off-centering of the Pb/Sn on the 4c Wyckoff site was observed.^19^ All the aforementioned XRD measurements were performed at room temperature, and in each case the crystal was determined to be the *Cmcm* space group. An ordered structure of Pb_2_SbS_2_I_3_ was determined by Doussier et al.^24^ by performing XRD measurements at 100 K. They observed a lowering in symmetry, due to the ordering of the cations along the cation–sulfur chains, to the monoclinic *P*2_1_/*c* space group.

Without knowledge of the underlying atomic structure of these materials, accurate predictions of the crystal properties and optoelectronic performance are impossible, hampering the development of this emerging material class. In this work, we investigate the various proposed crystal structures for Sn_2_SbS_2_I_3_ using a suite of computational techniques (including density functional theory, cluster expansion, and molecular dynamics) in combination with X-ray diffraction measurements to determine the true ground-state crystal structure of the A_2_BCh_2_X_3_ family. We extend our model to investigate temperature-dependent disorder in these materials, resolving the room-temperature structure and corresponding optoelectronic properties. Finally, we elucidate the impact of static and dynamic disorder on the electronic structure and highlight the many material properties shown by Sn_2_SbS_2_I_3_ associated with defect tolerance in lead halide perovskites.

## Computational Methodology

### Cluster Expansion and Monte Carlo Simulations

All density functional theory (DFT) calculations were performed using the projector augmented wave method as implemented in the Vienna ab initio simulation package (VASP).^27−31^ For geometry optimization the dispersion-corrected optB86b-vdW functional was chosen, as it accounts for the van der Waals interactions commonly observed in layered materials and has been shown to be important for obtaining accurate forces in these compounds.^22,32,33^ The plane-wave cutoff energy and k-mesh spacing were converged to a total energy difference of 1 meV/atom, with a 40% increase in plane-wave cutoff energy during relaxations to avoid Pulay stress effects. All combinations of cations along the M_2_S_2_ chains in 16 and 32 atom unit cells (55 structures) were generated using the integrated cluster expansion toolkit (ICET).^34−36^ The energies (normalized per atom) and bandgaps were calculated using the optB86b-vdW functional, and two separate cluster expansion (CE) models were fit to the data using ICET. The fitting used a pair-cluster cutoff of 12 Å, with the inclusion of triplet clusters having no influence. The CE was sampled using Monte Carlo (MC) simulations performed using the mchammer package under the canonical ensemble in a temperature range of 100–700 K.^34^ Simulations were run on a 3456 atom supercell for 10,000 equilibration steps, followed by 750,000 sampling steps. The cation ordering at each temperature was analyzed using the crystal-torture package.^37^ Further details of the convergence process can be found in the Supporting Information.

### Ab Initio Molecular Dynamics

Ab Initio Molecular Dynamics (AIMD) simulations were performed in VASP using a 128-atom supercell generated to match the cluster vectors of the structure obtained at 600 K with the MC simulation.^38^ The simulation was performed using the Nosé–Hoover thermostat in the NVT ensemble with a 2 fs time step. The optB86b-vdW functional was used with Γ-point only **k**-space sampling. Analysis of the AIMD trajectory depended on the pymatgen package.^39^

### Electronic Structure

For the calculation of the electronic band structure of the athermal ground-state crystal structure, the screened hybrid functional HSE06 was used with the inclusion of spin–orbit coupling (HSE06 + SOC) with the same converged plane-wave cutoff and *k*-point density.^40,41^ The shift current was calculated using the Wannier interpolation scheme as implemented in Wannier90 (further calculation detail provided in the Supporting Information).^42,43^ To determine the inverse participation ratio, the density of states was calculated using HSE06 for 1024-atom supercells matching the cluster vectors obtained from the MC simulations between 0 and 700 K.^38^ Electronic band structures were generated with sumo(44) (Figure S11) for ordered polymorphs, and easyunfold(45) was used to plot unfolded band structures for disordered structures (Figure S12). All calculation data and analyses are provided in an online repository at 10.5281/zenodo.7276405.

## Experimental Methodology

Sn_2_SbS_2_I_3_ single crystals were grown by chemical vapor transport using iodine as the transport agent. An evacuated and sealed quartz glass ampule containing the starting materials and the additional halogen (5 mg/cm^3^) was placed in a two-zone furnace with a temperature gradient from 450 to 350 °C. After a growth time of 240 h, needle-like crystals with a length of up to 10 mm and a diameter of 1–2 mm were obtained (Figure S1).

Single-crystal diffraction was performed on a Bruker APEX-II CCD diffractometer with Mo Kα radiation (λ = 0.71073 Å). Data acquisition was performed using Bruker APEX2, cell refinement and data reduction were performed using Bruker SAINT, and numerical absorption correction was performed using Bruker SADABS. Structure solution was performed with SHELXS97^46^ and refinement with SHELXL2018/3.^47^ Further details of the respective refinements may be found in the cif in the online repository. Guinier diffraction measurements were performed using a Huber Guinier diffractometer with Mo Kα_1_ radiation (λ = 1.54096 Å) and a closed-circle He-cooled cryosystem. Patterns were collected every 10 K in a heating cycle with cumulative measurement times of 600 s for every data point.

## Results and Discussion

### Computational Structural Analysis

To determine the ground-state crystal structure, the relaxed geometries and formation energies of 57 cation arrangements were calculated (all combinations of cations along the M_2_S_2_ in 16 and 32 atom unit cells plus the previously reported structures). Included in the set of structures were the experimentally determined configurations plus the theoretically predicted *Cmc*2_1_ structure. The relative DFT total energies of select structures are given in Table 1. In agreement with Kavanagh et al. the *Cmc*2_1_ structure is found to be more stable than the previously reported experimental structure. Although there is an increase in Madelung energy going from the ordered *Cmcm* polymorph to the ordered *Cmc*2_1_ polymorph, the structure is stabilized by an enhanced bonding interaction of the Sb^III^ cations with the anion p states brought about by the cation off-centering.^22^

**Table 1:** Relative Energies for the Proposed Crystal Structures of Sn_2_SbS_2_I_3_ as Calculated Using DFT (optB86b-vdW) without the Inclusion of Temperature Effects^a^

	*N* (unit cell)	MX_6_ polyhedron	M_2_Ch_2_ chain	relative energy (eV/atom)
*Cmcm* (Olivier-Fourcade et al.)^15^	16	Sb	-Sn-	0
*Cmc*2_1_ (Kavanagh et al.)^22^	16	Sb	-Sn-	–0.011
*Cmc*2_1_	16	Sn	-Sn-Sb-	–0.063
*P*2_1_/*c*	32	Sn	-Sn-Sn-Sb-Sb-	–0.071

a The experimental structure characterized by Olivier-Fourcade et al.^15^ has been set to 0 eV/atom.

A further energy lowering of 63 meV/atom is found by switching cation positions to a mixed Sn^0.5^Sb^0.5^ occupancy along the metal–sulfur chains. The lowest energy arrangement in a 16-atom cell is found when the Sn^II^ and Sb^III^ cations alternate along the M_2_Ch_2_ chain (ABAB) (Figure S2(b)). We also find an improvement in the agreement of the lattice parameters with experiment (Table S1), driven by a contraction of the metal–sulfur bonds along the chains. Electrostatics appear to be the main driving force in the reduction in energy with a 26 meV/atom reduction in the Madelung energy.

A further 8 meV/atom reduction in energy is found when the primitive cell is increased to 32 atoms due to a greater number of cation configurations being allowed. The lowest configuration is the centrosymmetric (antipolar) *P*2_1_/*c* space group where the cations are arranged in a zigzag -Sn-Sb-Sb-Sn- (ABBA) configuration along the metal–sulfur chains, as shown in Figure 1(c). In the *P*2_1_/*c* space group, the *a* vector is doubled and becomes the *c* vector. In the initial structures, the Sn(polyhedra) cations were centered between the metal–sulfur chains and became off-centered when the geometry was relaxed. The off-centering is present in the *Cmc*2_1_ (16-atom) cell; however the Sn(polyhedra)–S bond difference greatly increases from 0.06 Å in the *Cmc*2_1_ cell to 0.40 Å in the *P*2_1_/*c* (32-atom) cell, due to reduced Sn(polyhedra)–Sb(chain) repulsion facilitated by the ABBA chain ordering (Figure S3). This stabilizes the structure through an additional reduction in Madelung energy (Table S2) and an increase in bonding between the Sn(polyhedra) and neighboring anions.

A structure containing a -Sn-Sn-Sn-Sb-Sb-Sb- (AAABBB) configuration along the metal–sulfur chains was generated and was not found to be lower in energy, with the calculated DFT total energy lying 4 meV/atom above the ground state. The trained cluster expansion for Sn_2_SbS_2_I_3_ was used to screen all structures with a unit cell containing 48 or 64 atoms and found no lower energy polymorphs. The CE correctly predicted the energy of the -Sn-Sn-Sn-Sb-Sb-Sb- configuration in a 96-atom supercell, validating its predictive capabilities for cells larger than the training set and convergence with respect to cluster size (atomic interaction range), confirming that the *P*2_1_/*c* polymorph is the athermal ground state.

**Figure 2:**
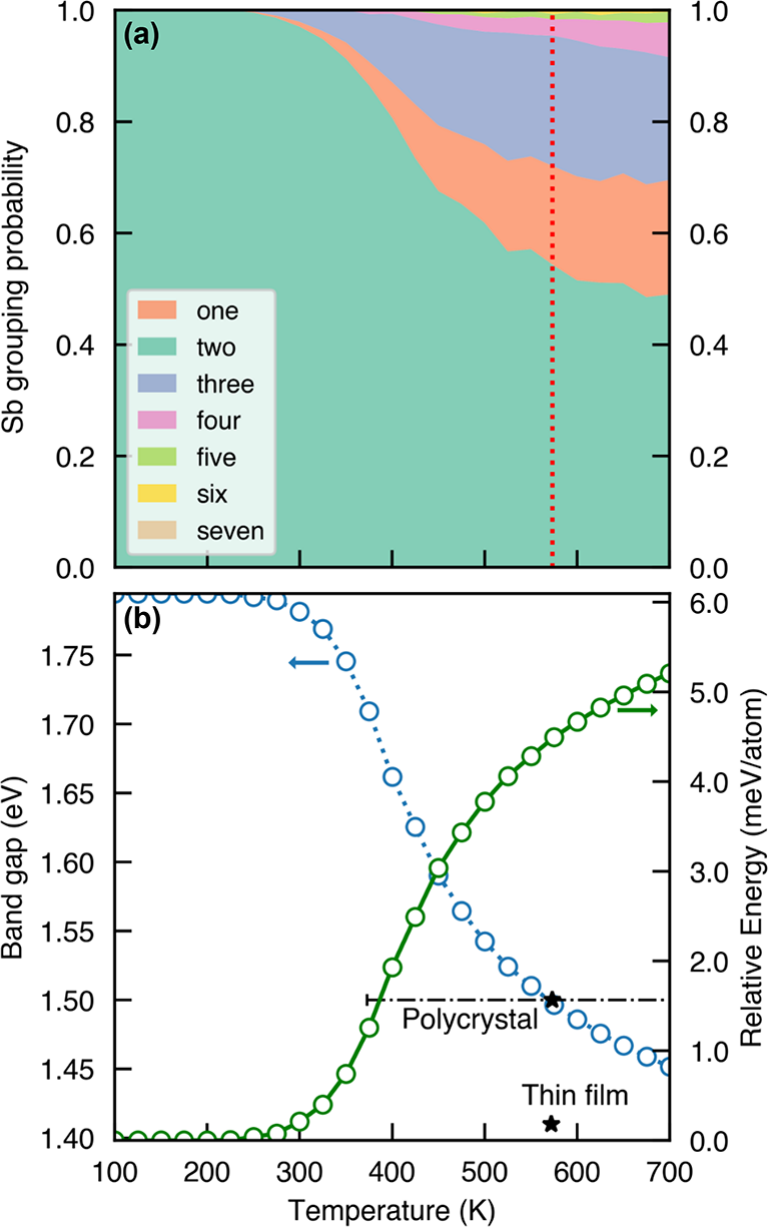
Temperature dependence of (a) Sb grouping sizes along the metal–sulfur chains, and (b) the calculated fundamental bandgap (blue) and relative internal energy (green) of Sn_2_SbS_2_I_3_. The red line is the thin film annealing temperature.^11^ Experimental bandgaps with respect to their synthesis temperatures are plotted as black stars, with the dash-dot line representing the temperature gradient used during the modified Bridgman method.^11,18^

To investigate how the crystal structure changes with temperature, a CE model trained using the structures with a mixed Sn^0.5^Sb^0.5^ occupancy along the metal–sulfur chains was sampled using MC simulations. In the ground state, the Sn^II^/Sb^III^ cations are grouped in pairs along the chain (ABBA); tracking deviations away from this ordering in Figure 2(a) shows that different arrangements of cations along the chains become accessible as temperature increases. The order–disorder transition occurs around 400 K and can be visualized by calculating the change in the heat capacity with increasing temperature, Figure S9. From Figure 2(a), we can see that at the annealing temperature of 573 K used by Nie et al.,^11^ over 40% of cations along the chains are no longer found in the ground-state configuration, compared to 75% at the fully random limit (*P*(*n*) = n*0.5^(*n*+1)^, where *n* is the size of the Sb cluster). The MC simulations predict that the fully random limit is not reached at temperatures below the melting point, determined to be 688.15 K by Starosta et al.^18^ Assuming that the arrangement of cations is frozen during cooling, this would suggest significant disorder is present in the final synthesized absorber.

**Figure 3:**
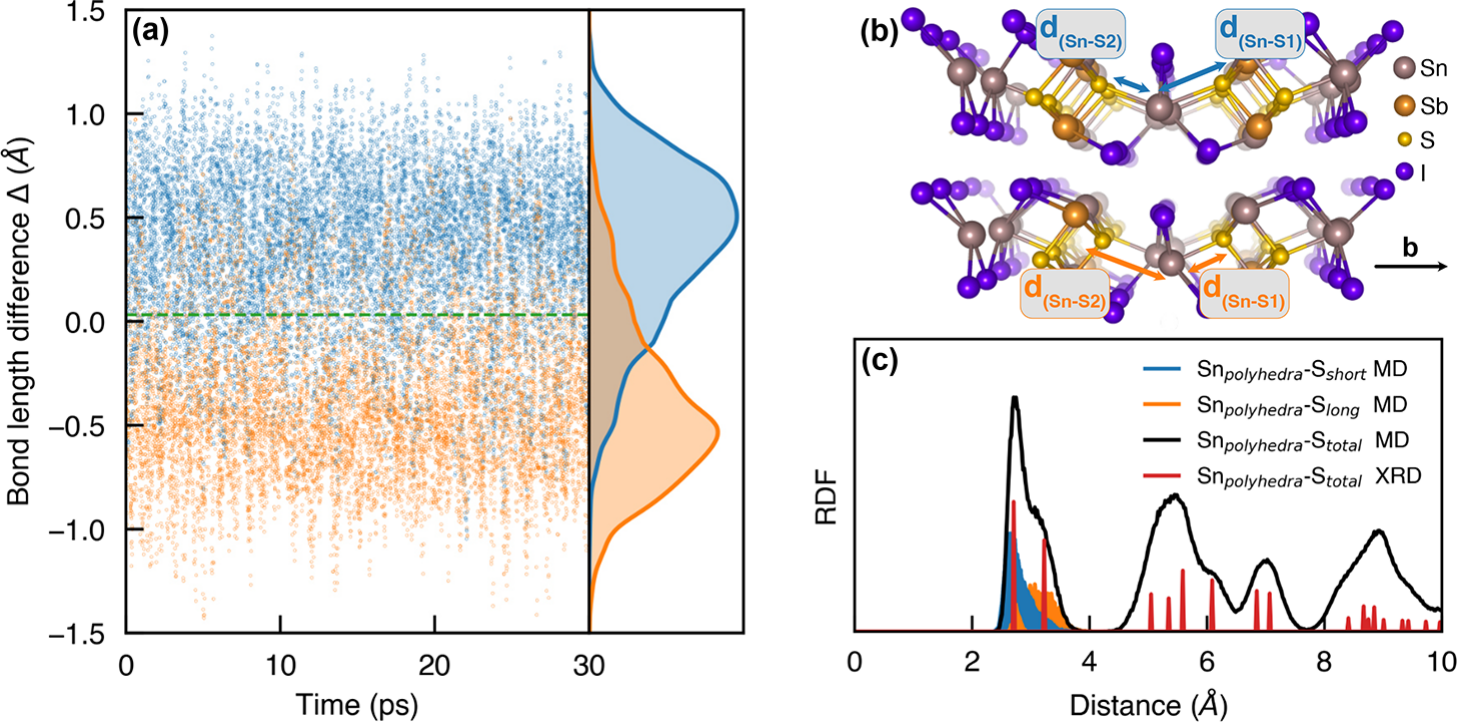
(a) Variation of the Sn(polyhedra)–S bond lengths in Sn_2_SbS_2_I_3_ at room temperature depicted through the bond length difference Δ = *d*_*Sn*–*S*1_ – *d*_*Sn*–*S*2_, where S1 is the sulfur neighbor in the +*b* direction. Configurations for which Δ is initially positive/negative are colored blue/orange to illustrate hopping behavior, with (b) showing an example of the assignment of S1/S2. (c) The radial distribution function (RDF) between Sn(polyhedra) and S sites.

Molecular dynamic simulations performed at 300 K provide insight into the difficulty in experimentally identifying the Sn^II^ off-centering from the *Cmcm*4c Wyckoff site. At 300 K there is significant thermal motion with swapping of the Sn(polyhedra)–S bond lengths occurring. Figure 3(a) plots the bond length difference, Δ = *d*_*Sn*–*S*1_ – *d*_*Sn*–*S*2_, where S1 is the sulfur neighbor in the +*b* direction. Averaging over each Sn(polyhedra) site in the 128-atom supercell during the MD simulation, the averaged Δ is close to zero. We would expect the averaged Δ to tend to zero with increasing supercell size; limitations on the size of the supercell during MD simulations prevents all possible cation arrangements predicted by the Monte Carlo simulations from being captured. This also explains why the density plots of the distribution of Δ values is close to, but not perfectly, symmetrical. The tailing of the densities into the other region shows significant hopping of the short/long bond, but that the Sn(polyhedra) is preferentially located near the starting position. This thermal motion of the cations results in the radial distribution function, Figure 3(c), being centered between the split Sn–S bond lengths, thus giving an apparently centered Sn position during XRD measurements. The average Sn(polyhedra)–S bond length of 2.99 Å agrees reasonably well with the Sb–S bond length reported by Ibanez et al. of 3.06 Å (where the polyhedron cation site was thought to be Sb). They also fitted a structure with inequivalent Sb(polyhedra)–S bond lengths, with a shorter bond of 2.72 Å and a longer bond measuring 3.23 Å. Again, the MD simulation agrees well with the experimental measurements, with an average short bond length of 2.77 Å and an average long bond length of 3.22 Å. These results show that cation disorder and thermal averaging are likely the reason behind experimental reports of the centrosymmetric *Cmcm* structure for this material.

### Experimental Structural Analysis

Given the limitations of previous crystal structure determinations, single crystals of Sn_2_SbS_2_I_3_ were grown through chemical vapor transport, and single-crystal diffraction was performed. To thoroughly probe the crystal symmetry, a full scan of the Ewald sphere was measured, with integration and data reduction in the monoclinic point group 2/*m* (corresponding to the C-centered space groups *C*2/*m* or *C*2/*c*) performed in order to avoid imposing symmetry onto the data. However, there was no evidence of symmetry reduction into the monoclinic system, and so we continued with structure refinement in the orthorhombic crystal system.

Initially, we found agreement with the previous crystal structure determinations, finding an orthorhombic crystal system in the centrosymmetric space group *Cmcm* with *a* = 4.2627(2) Å, *b* = 14.0341(8) Å, and *c* = 16.4293(10) Å. This is largely in accordance with Olivier-Fourcade et al.^15^ and Ibanez et al.,^16^Figure 1(a) and Figure S2(a). In agreement with the latter report, we find that the Sb site, nominally lying on the 4c Wyckoff position, is split and disordered above and below the mirror plane at *x*,*y*,1/4 and lies on 8f. This conventional model gave an excellent fit with both the refinement parameters (*R*_1_ = 2.0%, *wR*_2_ = 4.9%, GoF = 1.07) and the residual electron density (1.169 > Δ*e*^–^/Å^3^ > −1.478), suggesting a good agreement between data and model. One drawback, however, is the small difference in the structure factors between the isoelectronic cations Sn^2+^ and Sb^3+^, which means that great care has to be taken when comparing the different possible cation ordering motifs that can arise.

The observed disorder on the Sb position may be implied through the symmetry of the assumed space group, and it is, therefore, necessary to regard the possible subgroups that allow for ordering of the Sb position. Two possible subgroups allow for this distortion in different ways: (1) the polar space group *Cmc*2_1_ no longer contains a mirror plane in the (004) plane, Figure S4, and (2) the mirror plane is replaced by a glide plane in the centrosymmetric space group *P*2_1_/*c*, Figure S5.

The first case would give rise to a polar crystal structure, since all atoms on the Sb position would move in the same direction away from the (004) plane. When refining the single-crystal data against this model using one unique Sb position, however, the Sb atom does not move significantly away from the mirror plane, and large residual electron density peaks appear around the Sb position, suggesting an unaccounted degree of disorder. In fact, when reintroducing the split site through a supplementary Sb position on the supposedly empty site, Figure S4, one obtains a significantly better fit, suggesting that the structure at room temperature is not macroscopically polar.

**Figure 4:**
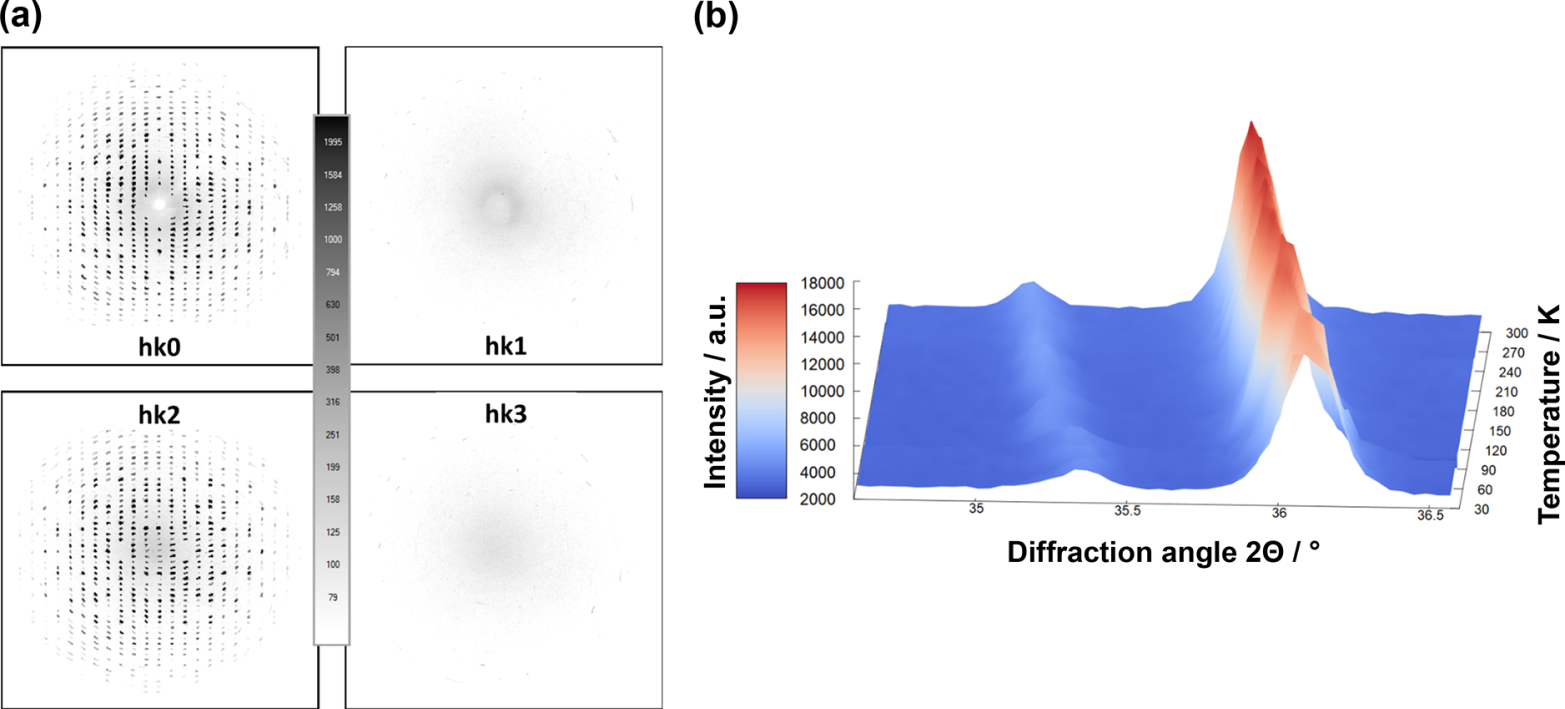
(a) Calculated precession images for the *hk*0, *hk*1, *hk*2, and *hk*3 layers indexed in the *P*2_1_/*c* crystal structure and (b) variable-temperature Guinier diffraction scans in the range 34.6–36.6° 2θ. Diffractograms were recorded every 10 K.

Since the space groups *Cmcm* and *Cmc*2_1_ share the same translational symmetry, potential experimental differences lie only in the diffraction intensities, while there are no supplementary reflections that could be detected. The same is not true for the transition from *Cmcm* to *P*2_1_/*c*. Notably, *a* in *Cmcm* becomes the *c* lattice parameter in *P*2_1_/*m* and is doubled during the klassengleiche symmetry descent from *P*2_1_/*m* to *P*2_1_/*c*, Figure S5. This doubling has the structural consequence that the atoms can shift away from the (004) plane alternatingly and would hence form an antipolar structure, in which the dipoles cancel each other out. Furthermore, the doubling would lead to supplementary reflections that can be observed in the odd *hk*1, *hk*3, *hk*5, etc., layers when indexing the diffraction data in this doubled lattice. Diffraction spots are clearly observed in the *hk*0 and *hk*2 planes, but there is no indication of Bragg diffraction in the *hk*1 and *hk*3 planes, Figure 4(a), suggesting that this ordering does not occur at room temperature. This analysis is independent of the cation swapping, indicating that the ordered *P*2_1_/*c* structure (athermal DFT ground state) is not present at room temperature.

Following this, we assessed the model predicted by our DFT and MC calculations, where the Sn and Sb cations are swapped, resulting in Sn residing on the split site (polyhedron), while the other cation position (chain) is shared between Sn and Sb. We find a slight improvement of the refinement parameters (*R*_1_ = 1.6%, *wR*_2_ = 3.6%, GoF = 1.13, corresponding to relative improvements of 5–27%), but more significantly, a smoother residual electron density (0.738 > Δ*e*^–^/Å^3^ > −0.974, a relative improvement of 35%) as compared to the conventional model (Sn_2_S_2_ chains rather than Sn_0.5_Sb_0.5_S_2_). In addition to these improvements in the refinement, we find the bond valence sums (BVS)^48,49^ for the cations in the conventional model are significantly too small for Sb^III^ (+1.90) and too large for Sn^II^ (+2.29). This means the bonds around the Sb position are longer than expected from the bond valence concept, while those involving Sn^II^ are shorter than expected. When Sn and Sb are switched, however, the BVS for Sb^III^ (+2.84) and Sn^II^ (+1.92 and +2.00) are much closer to their expected values (i.e., oxidation states). This notable difference is mainly due to the greater S coordination of highly charged Sb^III^ in the M_2_S_2_ chains (as opposed to the MI_6_ polyhedron) along with shorter Sb–S bonds than Sn–S bonds. Accumulating the refinement values and the BVS values, we can conclude with some confidence that our DFT+CE disordered *Cmcm* structure is indeed the room-temperature crystal structure for Sn_2_SbS_2_I_3_.

In the heavier homologue Pb_2_SbS_2_I_3_, one could attain a low-temperature phase transition between the disordered orthorhombic room-temperature structure and the antipolar ordered structure at lower temperatures.^24^ The group–subgroup relationship is given in Figure S5. In order to detect even small differences at lower temperatures, we conducted low-temperature Guinier diffraction concentrated on a specific range in the diffraction pattern, Figure 4(b). While the lower angle reflections would correspond to the 026 reflection in the orthorhombic *Cmcm* structure or the 160 reflection in the monoclinic *P*2_1_/*c* structure and would hence not split, the stronger reflection corresponds to the 134 reflection in the orthorhombic crystal structure and should split into the −242 and 142 reflections in the monoclinic structure, which should be roughly of equal intensity. There is, however, no sign of such splitting, indicating that the Sn^II^/Sb^III^ disorder introduced during sample preparation is static. This suggests that once the crystal has been synthesized, the energy barrier for cation rearrangement along the metal–sulfur chains is too large to permit rearrangement to the predicted athermal ground-state structure at room temperature.

### Impact of Disorder on the Electronic Structure

The calculated (HSE06+SOC) bandgap of the *P*2_1_/*c* athermal ground-state crystal is 1.78 eV, as shown in Figure S11, which is significantly larger than the experimentally determined optical bandgap of 1.4–1.5 eV.^11,18^ However, our CE model and MD simulations demonstrate significant cation disorder in these materials, which has been shown to markedly impact the optical properties of photovoltaic absorbers.^6,50−52^ To investigate the relationship between bandgap and atomic disorder, the electronic bandgap of the training structures was calculated using the optB86b-vdW functional on which a CE model was then trained. A scissor operator of +0.45 eV was applied to the results from the MC sampling of the CE model due to the tendency for GGA functionals to underestimate the bandgap. This scaling was validated by calculating the DFT bandgaps of 1024-atom supercells corresponding to MC cluster vectors from *T* = 200 to 700 K, showing close matches to the scaled results, Δ*E*_g_ = 0.02 eV, with further discussion in the Supporting Information. In Figure 2(b), we see the typical trend of increasing temperature and therefore increasing atomic disorder resulting in a reduction of the bandgap energies of Sn_2_SbS_2_I_3_. We find a dramatic reduction in the bandgap from 1.8 eV at *T* < 300 K to below 1.45 eV at *T* = 700 K.

Employing the frozen atom model (which assumes that atomic disorder is “frozen in” during the elevated temperatures of crystal synthesis and annealing), our model predicts a bandgap of 1.50 eV at the experimental annealing temperature of 573 K used during the fabrication of thin film devices (Figure 2), compared to the experimentally measured optical bandgap of 1.41 eV.^11^ Comparison to the optical bandgap of 1.5 eV measured by Starosta et al.^18^ using photoconductivity measurements is more challenging due to their chosen synthesis method. Oriented ingots were obtained using a modified Bridgman method in which an ampule, heated to 773.15 K, was pulled across a 400 K/cm temperature gradient to form ingots of length 50–60 mm. Our model at 570 K agrees best with the optical bandgap measurement of 1.5 eV, deviating between −0.05 and +0.2 eV across the temperature gradient.

Our results show that static disorder is the major contributor to the reduction in the bandgap; however, it alone cannot account for the total reduction in the size of the bandgap. During the molecular dynamics simulation, which investigated dynamic disorder in Sn_2_SbS_2_I_3_, the bandgap was found to fluctuate by up to ±0.175 eV. The fluctuations, in combination with changes to the electronic structure driven by sample inhomogeneity from the solution synthesis, may reduce the bandgap further.^53,54^

The results demonstrate the sensitivity of the electronic bandgap to static cation disorder in these materials, potentially allowing for the optimization of the material properties for photovoltaic operations. Cation disorder can be induced at relatively low temperatures (for example compared to the widely investigated II–IV–N_2_ nitrides with typical order–disorder transitions in the range 2000–3000 K), allowing for disorder engineering under less energy-intensive conditions.^55−57^

**Figure 5:**
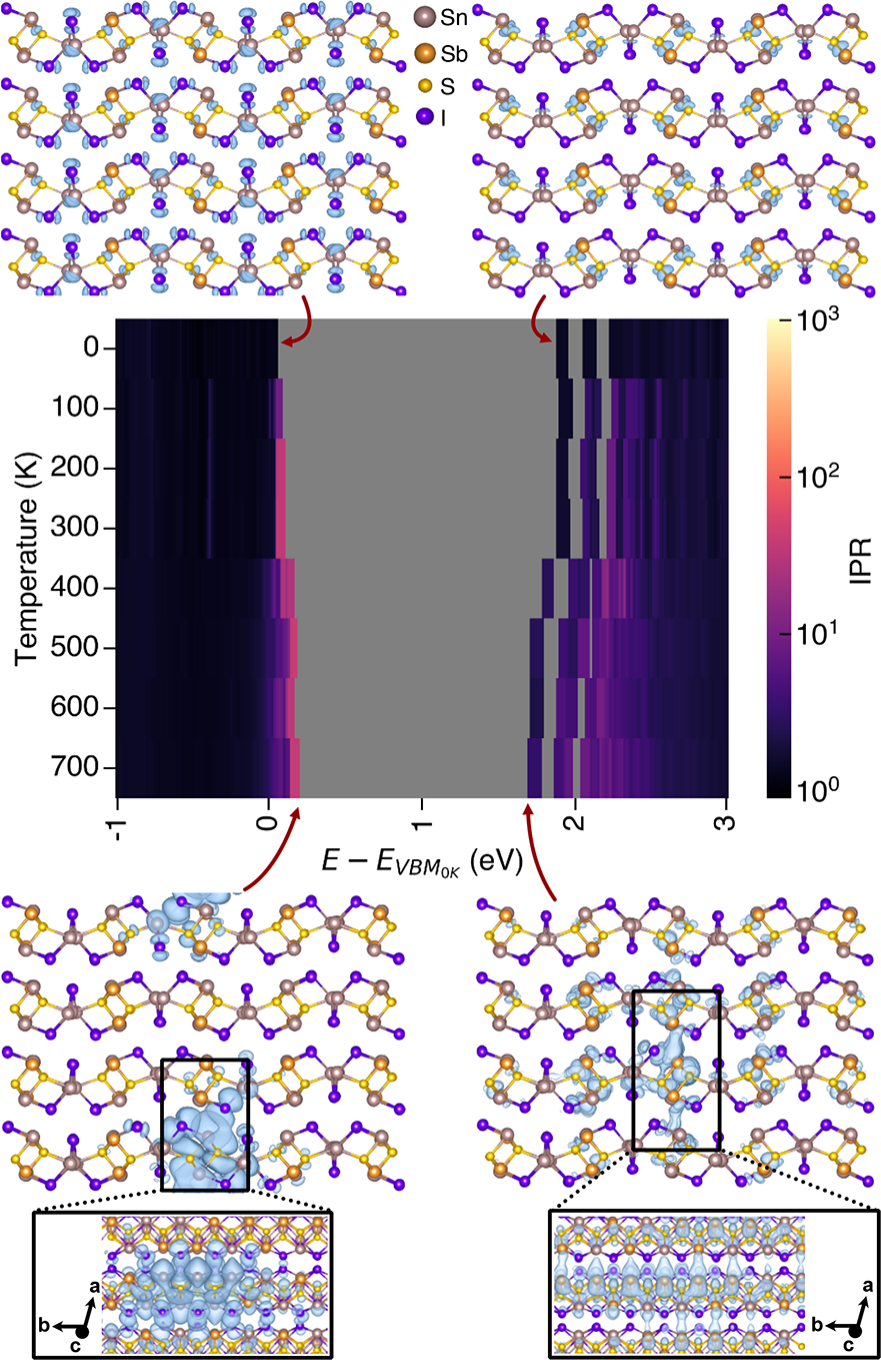
IPR of Sn_2_Sb_2_SI_3_ between 0 and 700 K, with the charge density isosurfaces (blue) of the VBM and CBM plotted for 0 and 700 K. Isovalue set to 5 × 10^–4^ e/Å^3^, and the atom colors are as follows: Sn = beige, Sb = orange, S = yellow, I = purple.

To study what factors are contributing to the change in the bandgap, we calculated the inverse participation ratio (IPR)^56,57^ of the electronic states in 1024-atom supercells matching the cluster vectors obtained from the MC simulations between 0 and 700 K. The IPR is obtained from the local density of states,
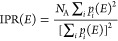
1where *N*_A_ is the number of atoms in the supercell and *p*_*i*_ is the local density of states for each atom *i*, calculated using the HSE06 functional. An IPR of 1 indicates that an electronic state is fully delocalized, while a value of 1024 (= *N*_A_) corresponds to localization on a single atom. To compare the IPR of different cation configurations, the energies were aligned using the core states. Figure 5 shows that as temperature increases, the valence band maxima (VBM) and the conduction band maxima (CBM) both shift, decreasing the bandgap, with a maximum IPR of 40 obtained at the VBM at *T* = 700 K. This increase in localization at the VBM can hamper carrier transport. From the charge densities, the VBM goes from being composed of Sn s–I p interactions in the metal-halide polyhedras to being localized around the longest grouping of Sn along the M_2_S_2_ chains at 700 K (Sn s, S p, and I p). The decrease in the CBM energy is less gradual, with a large reduction at 400 K coinciding with the order–disorder phase transition. The charge densities show a slight increase in localization on Sb sites; however, this is significantly reduced in comparison to the VBM, with a max IPR of 3 at 700 K. The increase in localization can also be seen in the flat VBM of the unfolded band structure of a 600 K disordered supercell (Figure S12). It is of note that no defect states are formed in the bandgap, in contrast to other cation-disordered chalcogenides and pnictides,^54,56,57^ indicating the disorder in this system may not accelerate nonradiative charge-carrier recombination—a promising sign for photovoltaic operation.

From the unfolded band structure shown in Figure S12, we find that cation disorder does not significantly impact the band edge dispersion, with minimal changes to electron effective masses, Table S5. Combined with the lack of shallow localized states in the bandgap, which can trap carriers,^54,56,57^ we would not expect changes to the band structure to reduce carrier mobility significantly for this material. The disorder will introduce cation disorder scattering due to the aperiodic potential originating from the valence difference; however this will be limited by the large static dielectric constant (ϵ_*xx*,*yy*,*zz*,*xz*_^0^ = [12.63, 49.20, 51.07, −12.15]).^58^

As well as showing signs of “disorder tolerance”, with no major increase in localization or reduction in carrier masses with disorder, Sn_2_SbS_2_I_3_ also exhibits several properties associated with defect tolerance in lead-halide perovskites. We find the VBM to have antibonding character due to the orbital repulsion between the Sn s^2^ and anion p states, illustrated by the crystal orbital Hamiltonian population analysis shown in Figure S13. An antibonding valence band edge is associated with the formation of *shallow* acceptor defects, which do not contribute to electron–hole recombination.^3,59,60^ Furthermore, Sn_2_SbS_2_I_3_ has relatively small electron effective masses (0.2–0.6 *m*_0_; Table S5), which combined with a large static dielectric constant, will limit the Coulomb interaction between charge carriers and defect sites, further reducing the probability of carrier trapping and recombination.^61^ Such defect tolerance is also supported by the lack of localized states formed in the bandgap when disorder is introduced, in contract to other disordered chalcogenides without these defect tolerant properties,^54^ as well as by the experimentally measured long photoluminescence lifetime (>7 ns).^11^ Additionally, photovoltaic absorbers with chain-like structures, such as Sb_2_Se_3_ and BiOI, have seen recent interest as the structure increases the likelihood of benign grain boundaries forming, reducing a source of recombination.^62,63^ We expect these properties to also be present in the other unexplored members of the A_2_BCh_2_X_2_ family, due to their chemical similarities.

We further assessed the effect of the absence of inversion symmetry on the photocurrent by calculating the shift current. Although the ground-state *P*2_1_/*c* is centrosymmetric and does not have a second-order optical response, the *Cmc*2_1_ phase (8 meV/atom above *P*2_1_/*c*) exhibited a sizable shift current tensor as shown in Figure S14. Furthermore, within 2.0 meV/atom above the *P*2_1_/*c* phase, we identified two *Cm* phases that exhibited comparable shift currents with the *Cmc*2_1_ phase, Figure S15. As the *Cm* phases have low enough energy, they may be realizable depending on the synthesis conditions. The performance of Sn_2_SbS_2_I_3_ photovoltaics could benefit from the optimal bandgap size and an enhancement from the non-centrosymmetric structure.

In conclusion, we determined the athermal ground-state structure of Sn_2_SbS_2_I_3_ to be the *P*2_1_/*c* space group. We find significant cation disordering between 300 and 900 K and confirm the predicted disordered *Cmcm* room-temperature structure using X-ray diffraction. Disorder is found to drastically affect the electronic bandgap, while avoiding the detrimental presence of localized states. By controlling the crystal annealing temperature as well as the subsequent crystal cooling rate, the disorder and thus bandgap energies of this and potentially other A_2_BCh_2_X_3_ materials could be finely tuned, allowing the targeted application of these emerging earth-abundant absorber materials. The predicted disorder and defect tolerance would allow the design of a graded solar cell which avoids the formation of recombination sites at crystal boundaries due to variations in structure. Therefore, the minimally investigated group IV/V mixed-metal chalcohalide family (A = Pb, Sn, Ge; B = Sb, Bi; Ch = O, S, Se; X = I, Br, Cl) presents an exciting class of materials for optoelectronic applications.

## Data Availability

All calculation data and analyses are provided in an online repository at 10.5281/zenodo.7276405.

## Supporting Information Available

Additional crystal structures, group–subgroup relationships, cluster expansion and Monte Carlo convergence plots, and further discussion of the electronic structure (PDF)
