# Supplementary material for: Interplay of Static and Dynamic Disorder in the Mixed-Metal Chalcohalide Sn2SbS2I3
Source: J Am Chem Soc. 2023 May 30;145(23):12509–17. doi: 10.1021/jacs.2c13336 (PMC10273231; doi:10.1021/jacs.2c13336)
Supplement: Supplementary file 1 — ja2c13336_si_001.pdf [file ja2c13336_si_001.pdf]

Supporting Information: Interplay of static and dynamic disorder in the mixed-metal chalcohalide $Sn_2SbS_2I_3$

Adair Nicolson,[†] Joachim Breternitz,[‡] Seán R. Kavanagh,[†,¶] Yvonne Tomm,[‡] Kazuki Morita,[¶,||] Alexander G. Squires,[†] Michael Tovar,[‡] Aron Walsh,[¶] Susan Schorr,[‡,§] and David O. Scanlon[*,†]

†*Thomas Young Centre and Department of Chemistry, University College London, 20 Gordon Street, London WC1H 0AJ, U.K.*

‡*Helmholtz-Zentrum Berlin für Materialien und Energie, Structure and Dynamics of Energy Materials, Hahn-Meitner Platz 1, 14109 Berlin, Germany*

¶*Thomas Young Centre and Department of Materials, Imperial College London, Exhibition Road, London SW7 2AZ, U.K.*

§*Freie Universität Berlin, Department of Geosciences, Malteserstr. 74-100, 12249 Berlin, Germany.*

||*University of Pennsylvania, Department of Chemistry, 231 S. 34 Street, Philadelphia 19104-6323, USA*

E-mail: d.scanlon@ucl.ac.uk

Crystal Growth

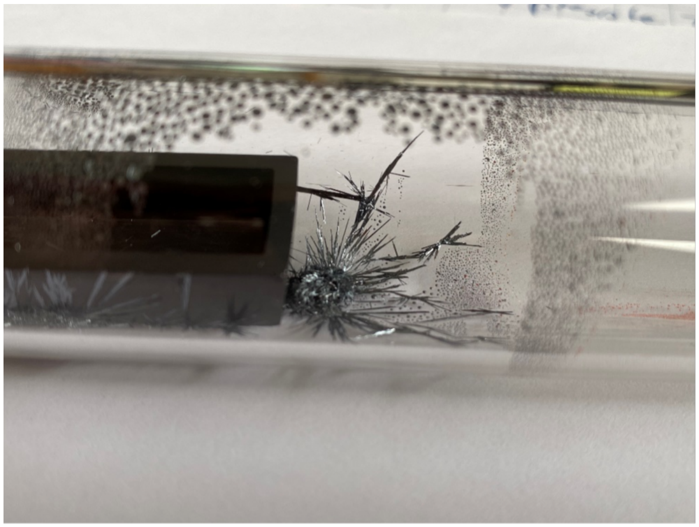

Figure S1: $Sn_2SbS_2I_3$ crystals grown through chemical vapour transport.

Crystal structures

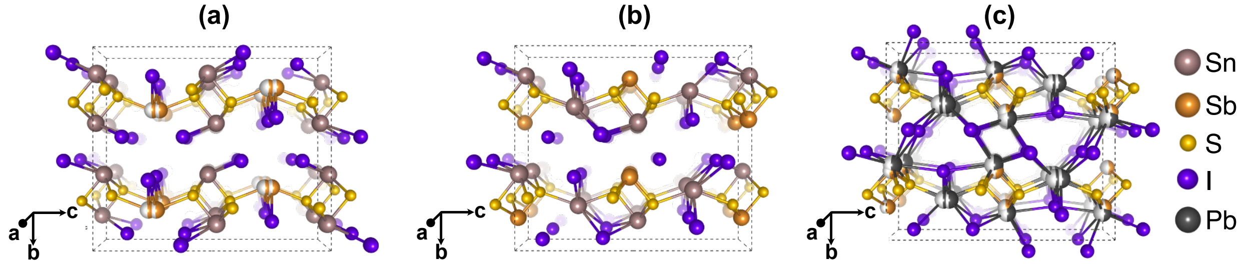

Figure S2: The experimentally determined room temperature conventional unit cell of (a) $Sn_2SbS_2I_3$ from Ibanez et al.,[S1] (b) the lowest energy 16 atom $Sn_2SbS_2I_3$ configuration, and (c) the room temperature experimental structure of $Pb_2SbS_2I_3$.[S2] The atom colours are as follows: Sn = beige, Sb = orange, S = purple, I = purple, Pb = grey. Half-coloured spheres represent atomic sites with 50% occupancy. All figures generated using `VESTA`.[S3]

Table S1: Experimental and calculated lattice parameters of the conventional unit cell for select $Sn_2SbS_2I_3$ structures. In the $P2_1/c$ structure the a lattice vector doubles and becomes c, and the c lattice vectors becomes b.

Structure	a/Å	b/Å	c/Å	Volume/Å^3
$Cmcm$ (Exp Olivier-Fourcade et al.)[S4]	4.28	14.05	16.47	989.58
$Cmc2_1$ (Kavanagh et al.)[S5]	4.29	14.31	16.51	1013.8
$Cmc2_1$	4.26	14.15	16.37	985.91
$P2_1/c$	7.35	16.49	8.55	992.04

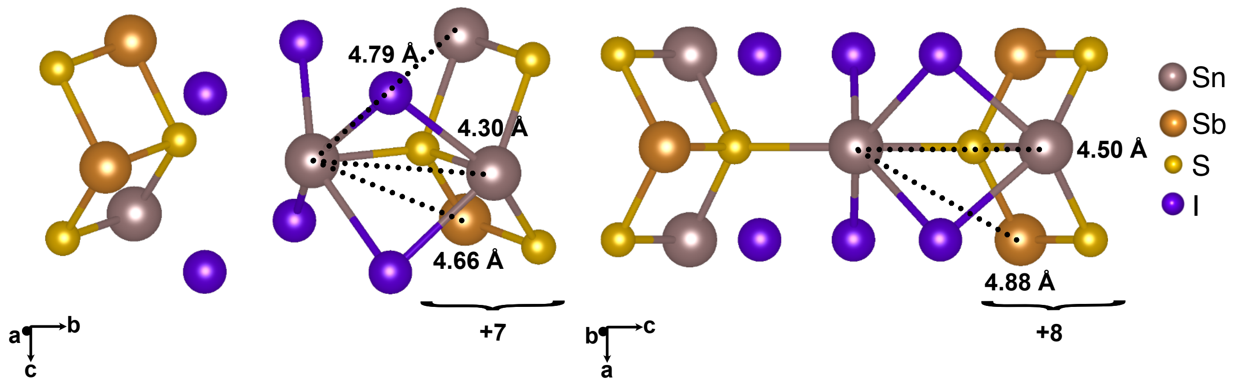

Figure S3: The distances between the Sn(polyhedra) and nearest cations for a) $P2_1/c$ and b) $Cmc2_1$. Sum of charges along the metal-sulfur chains given. The atom colours are as follows: Sn = beige, Sb = orange, S = purple, I = purple, Pb = grey. Half-coloured spheres represent atomic sites with 50% occupancy. All figures generated using `VESTA`.[S3]

Table S2: Madelung energies calculated, using the Mulliken potentials determined by `LOBSTER`,[S6,S7] for the proposed crystal structures of $Sn_2SbS_2I_3$. The lowest energy structure with cation disorder for a 16 and 32 atom primitive cell are included.

Structure	Madelung energy (eV/atom)
$Cmcm$ (Olivier-Fourcade et al.)[S4]	-0.820
$Cmc2_1$ (Kavanagh et al.)[S5]	-0.804
$Cmc2_1$	-0.830
$P2_1/c$	-0.834

Symmetry Relationships

8f Sn	8f 50%Sb	8f S	8f I1	4c I2
0	0	0	0	0
0.134	0.201	0.273	0.441	0.144
0.482	0.767	0.580	0.378	¼

C 2/m 2/c 2_1/*m*

t2

C m c 2_1

4a Sn	4a Sn′	4a Sb	4a □	4a S	4a S′	4a I1	4a I1′	4a I2
0	0	0	0	0	0	0	0	0
0.134	0.134	0.201	0.201	0.273	0.273	0.441	0.441	0.144
0.482	0.018	0.767	0.733	0.580	0.920	0.378	0.122	0.25

Figure S4: Group-Subgroup relationship between the conventional centrosymmetric crystal structure with split Sb site in the *Cmcm* space group and its polar subgroup $Cmc2_1$.

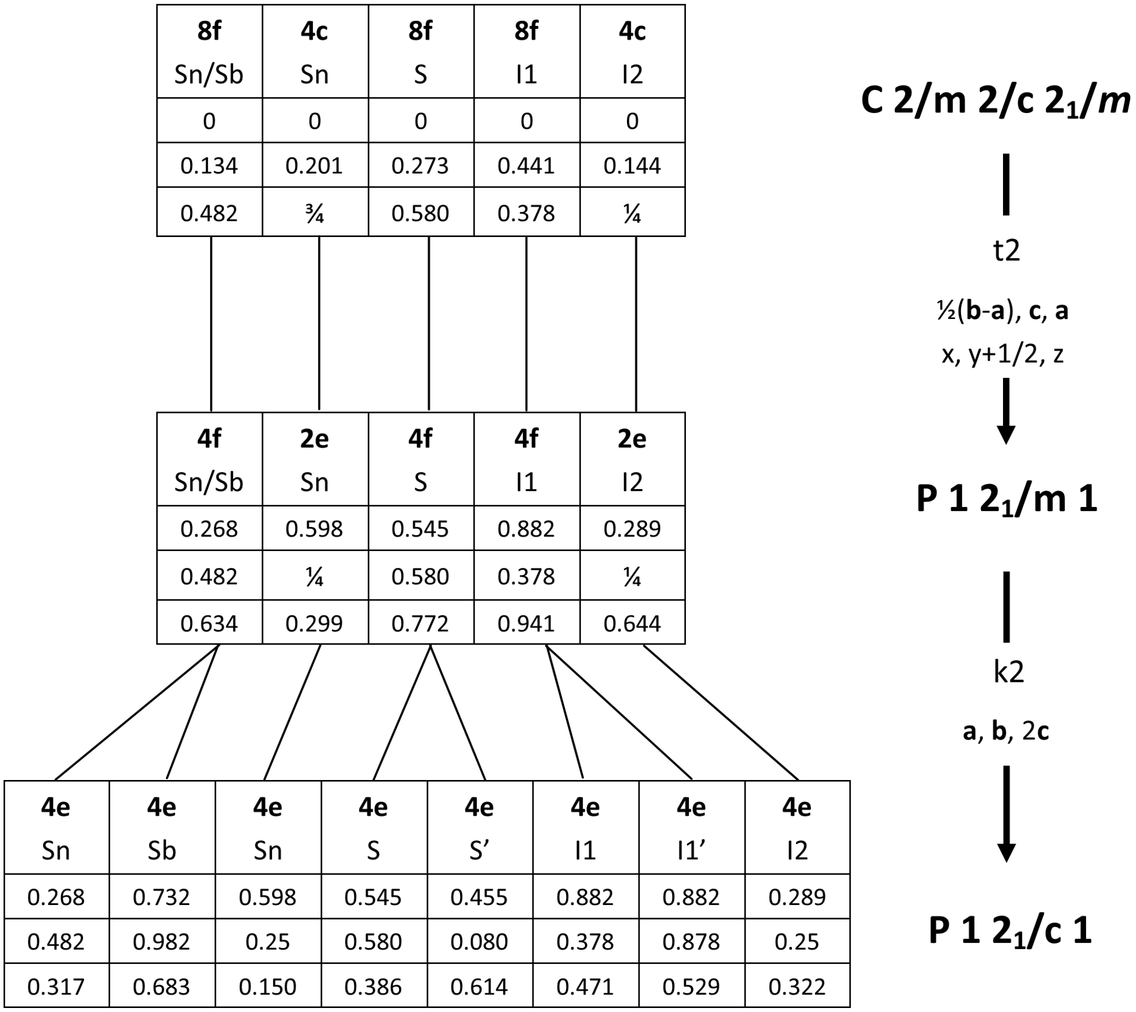

Figure S5: Group-subgroup relationship between the room temperature disordered crystal structure in the space group $Cmcm$ and the subgroup $P2_1/c$. We note that the intermediate space group $P2_1/m$ is not experimentally observed, but links group and subgroup.

Cluster Expansion and Monte Carlo

The cluster expansion (CE) was trained using the least absolute shrinkage and selection operator (lasso) method with a pair cutoff radius of 12 Å, with the inclusion of triplet clusters having no appreciable influence. Convergence was tested with respect to cutoff radius, Figure S7, and training data size, Figure S8. The predictive error for the cluster expansion is given in Figure S6.

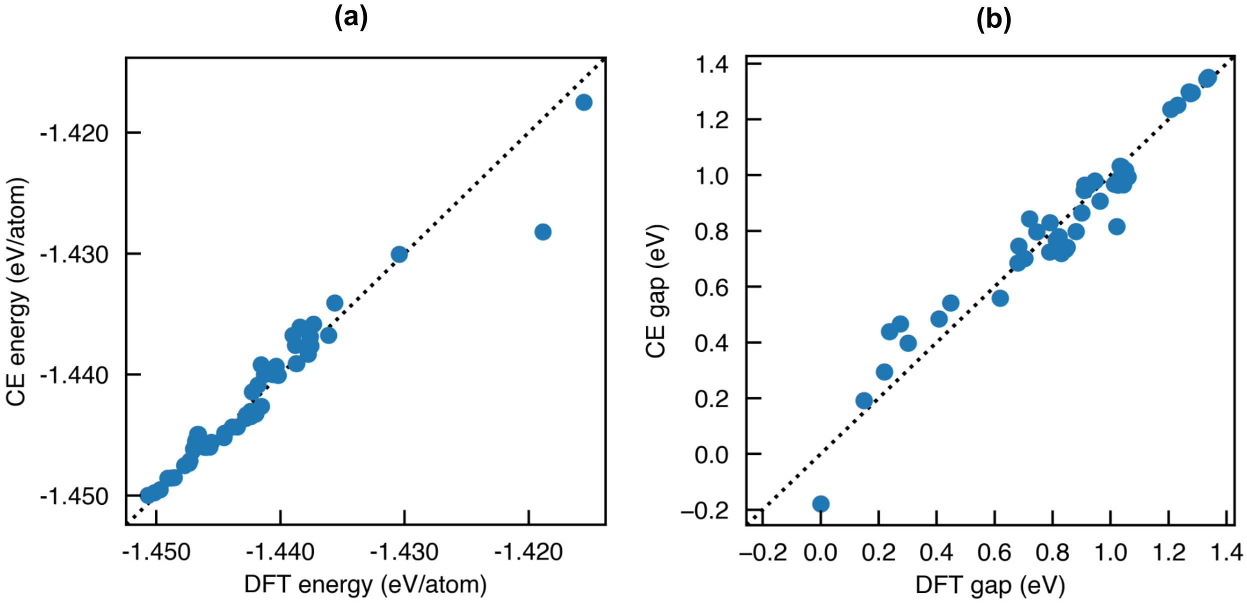

Figure S6: Predictive error for the $Sn_2SbS_2I_3$ cluster expansion for **(a)** the internal energy and **(b)** the band gap.

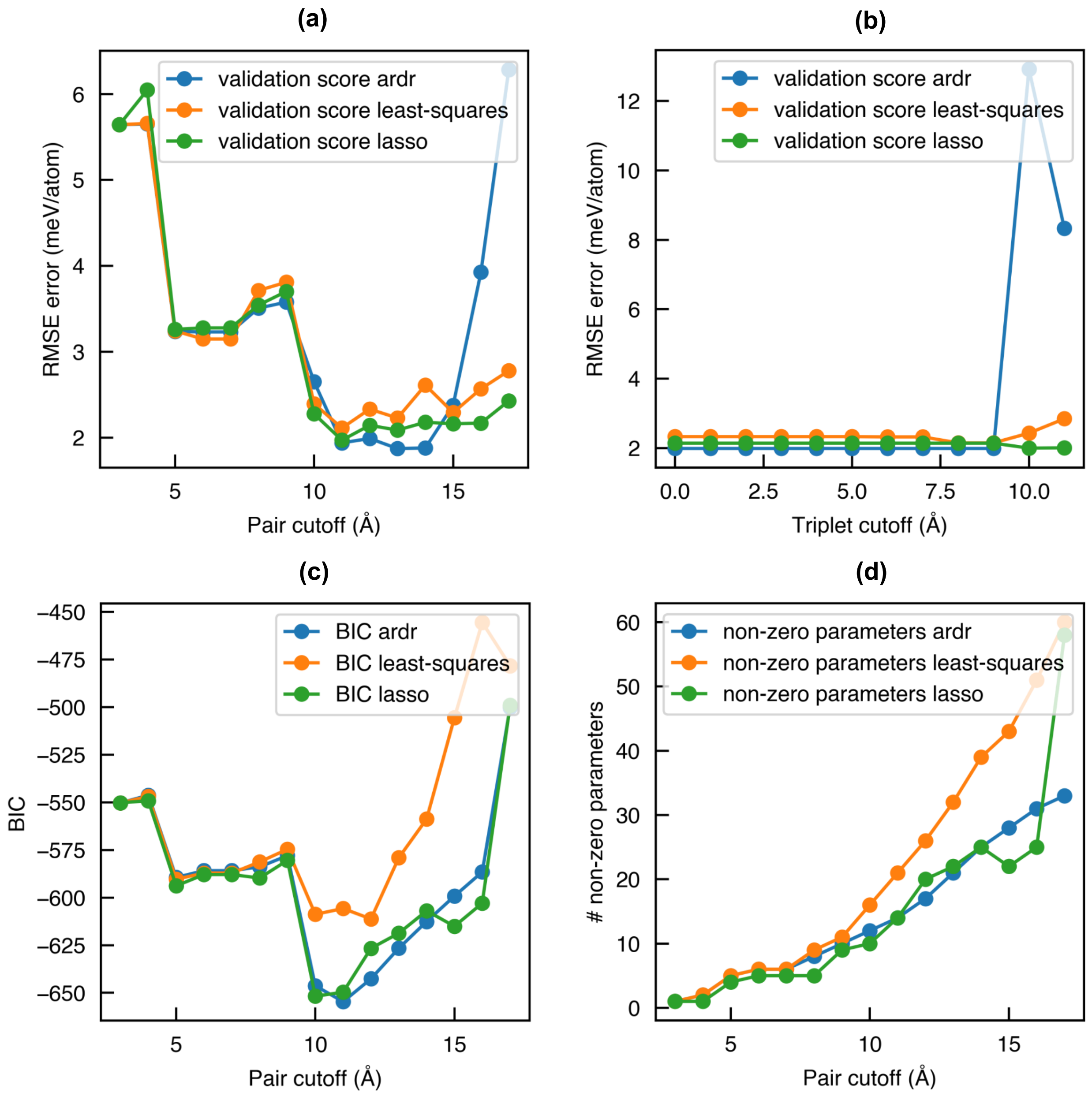

Figure S7: Learning curves showing the k-fold cross-validated root-mean-square error dependence on **(a)** doublet and **(b)** triplet cut-off radius. **(c)** Plots the dependence of bayesian information criterion and **(d)** the number of non-zero parameters on the pair cutoff length.

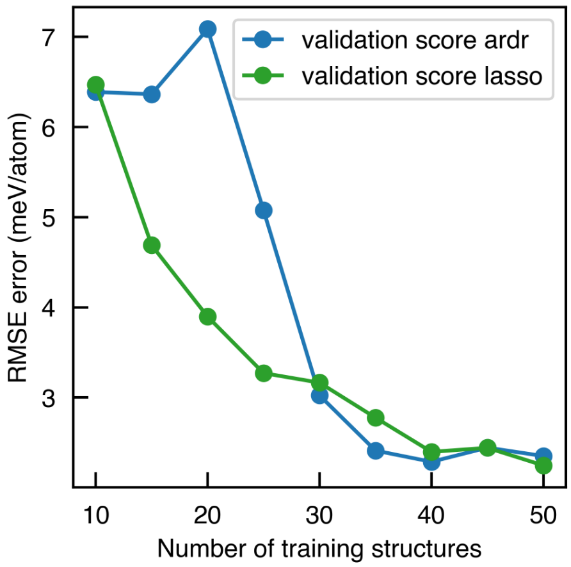

Figure S8: Learning curves showing the k-fold-validated root-mean-square error dependence on training set size.

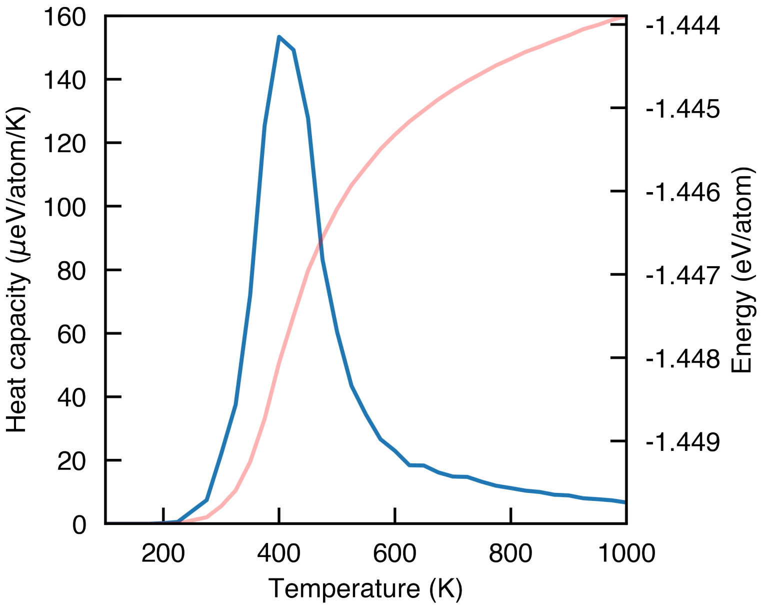

Figure S9: The change in heat capacity from the MC simulations with respect to temperature is shown by the blue line. The pale red line is the change in energy of the structure.

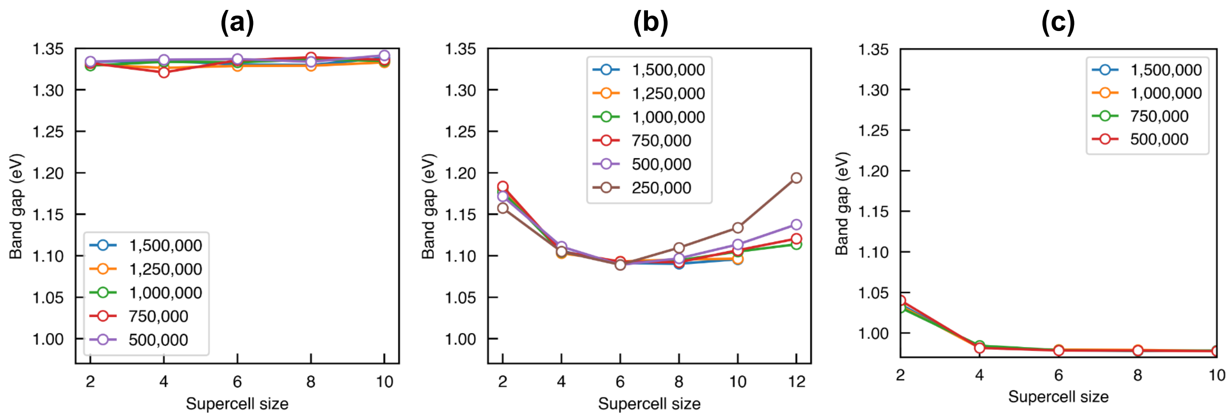

Figure S10: Convergence of the band gap from Monte Carlo simulations with respect to supercell size and number of simulation steps at **(a)** 300K, **(b)** 500K, and **(c)** 800K.

Additional Electronic Analysis

The electronic band structure for the athermal ground-state of $Sn_2SbS_2I_3$ was determined using the optB86b-vdW functional, HSE06 and HSE06 + spin-orbit coupling (Figure S11). In each case, the CBM was positioned at **Y** and the VBM located along the $\mathbf{\Gamma} \rightarrow \mathbf{Z}$ high-symmetry path. The structure is found to have an indirect gap, with a difference of 0.07 eV between the indirect and direct gap.

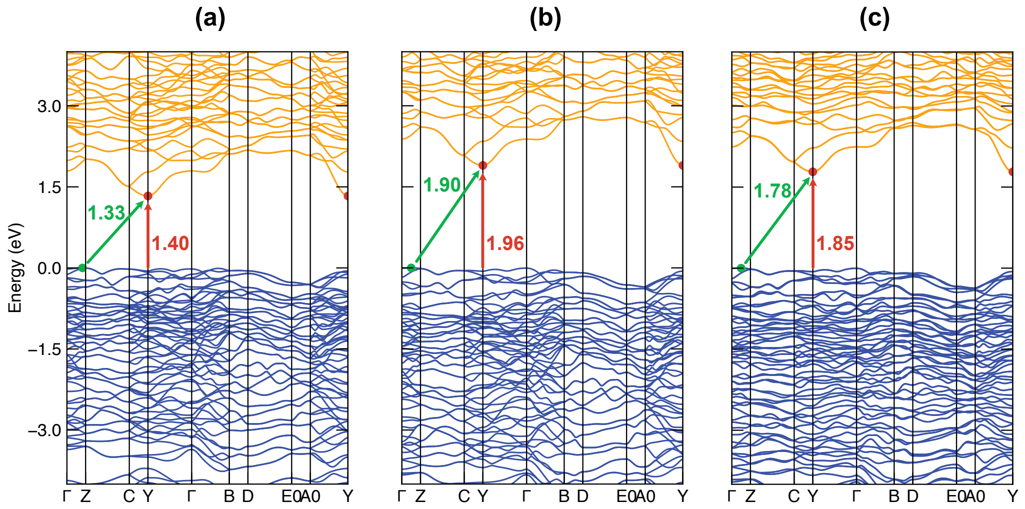

Figure S11: Electronic band structure for the athermal ground-state of $Sn_2SbS_2I_3$ using **(a)** the optB86b-vdW functional, **(b)** HSE06 and **(c)** HSE06 + spin-orbit coupling. Valence band in blue, conduction band in orange. VBM set to 0 eV. All figures generated using `sumo`.[S8]

To investigate the relationship between bandgap and atomic disorder, the electronic bandgaps of the training structures were calculated using the optB86b-vdW functional,[S9] on which a CE model was then trained using these calculated bandgaps. The predicted band gaps were converged with respect to supercell size and the number of MC steps at 300K, 500K and 800K (Figure S10). To account for the tendency of GGA functionals to underestimate the band gap, the results from the MC sampling were scaled using the band structure calculation of the athermal ground-state. Two different scaling methods were tested. The first was a linear scaling:[S10] $E_g = E_g(CE) * E_g(HSE06 + SOC)/E_g(optB86b - vdW) = E_g(CE) * 1.34$ and the second, a scissor operator:[S11] $E_g = E_g(CE) + [E_g(HSE06 + SOC) - E_g(optB86b -$

$vdW)] = E_g(CE) + 0.45eV$.

Table S3: Calculated band gap for supercells of different sizes with cluster vectors matching those obtained from MC simulations at 600 K. The band gap for the 3456 atom supercell is the result from the MC simulation.

	128 atoms	1024 atoms	3456 atoms (MC simulation)
E_g(optB86b-vdW)	1.16 eV	1.09 eV	1.04 eV
E_g(HSE06)	1.71 eV	1.64 eV	-
E_g(HSE06 + SOC)	1.61 eV	-	-

The validity of these scaling techniques was tested by calculating the band gap of various supercells of different size whose cluster vectors matched those obtained from the MC simulation at 600 K, these results are presented in Table S3. The difference in band gap energy when switching functionals is consistent between supercell size and when compared to the athermal ground state (Figure S11). We find the scissor operator provides the best match when comparing the MC simulated band gap (1.04 eV shifted to 1.49 eV) with the 1024 atom DFT calculated band gap (1.64 eV shifted to 1.54 eV) when including a further reduction of the band gap by 0.1 eV to account for spin orbit coupling effects. We also find good agreement at other temperatures (Table S4). The remaining discrepancy can be explained by the inability for the 1024 atom supercell to exactly replicate the cluster vectors present in the 3456 atom supercell used during the MC simulations.

Table S4: Calculated HSE06 band gaps for 1024 atom supercell whose cluster vectors match those obtained from MC simulations in the temperature range 200-700 K. The HSE06 band gaps have been shifted by -0.1 eV to account for spin orbit coupling. The MC band gaps have been shifted by +0.45 eV to account for GGA functionals underestimating the band gap of semiconductors.

	200 K	300 K	400 K	500 k	600 K	700 K
E_g(HSE06)	1.86 eV	1.86 eV	1.70 eV	1.63 eV	1.64 eV	1.57 eV
E_g(HSE06) − 0.10 eV	1.76 eV	1.76 eV	1.60 eV	1.53 eV	1.54 eV	1.47 eV
Monte Carlo + 0.45 eV	1.79 eV	1.76 eV	1.59 eV	1.53 eV	1.49 eV	1.45 eV

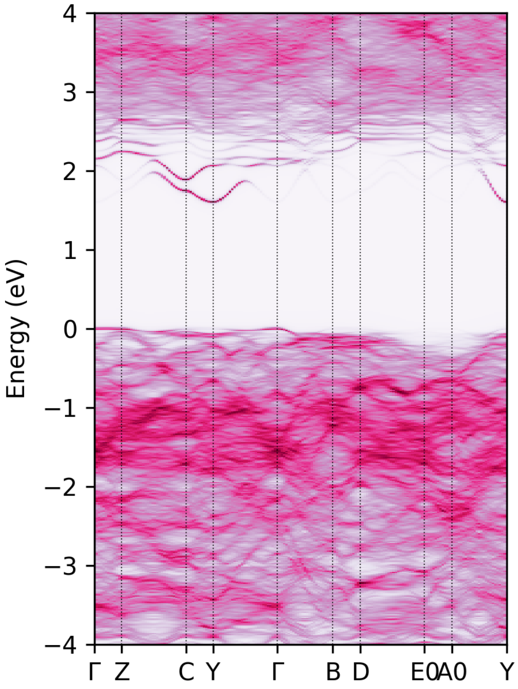

Figure S12: Unfolded electronic band structure for a 128 atom supercell whose cluster vectors match those obtained from the MC simulation at 600 K. The band structure was unfolded using `easyunfold`.[S12]

Table S5: Carrier effective masses for the athermal ground-state of $Sn_2SbS_2I_3$ and a 128 atom supercell whose cluster vectors match those obtained from the MC simulation at 600 K. Calculated using HSE06 hybrid DFT with spin orbit coupling. In the athermal ground state, the VBM is located along the $\mathbf{\Gamma} \rightarrow \mathbf{Z}$ high-symmetry path.

Athermal ground state $P2_1/c$				
Hole (m_0)		Electron (m_0)		
VBM→Z	VBM→Γ	Y→C	Y→Γ	Y→A0
2.00	5.85	0.62	0.33	0.20

Disordered supercell 600 K				
Hole (m_0)		Electron (m_0)		
C→Z	C→Y	Y→C	Y→Γ	Y→A0
1.18	3.96	0.61	0.31	0.22

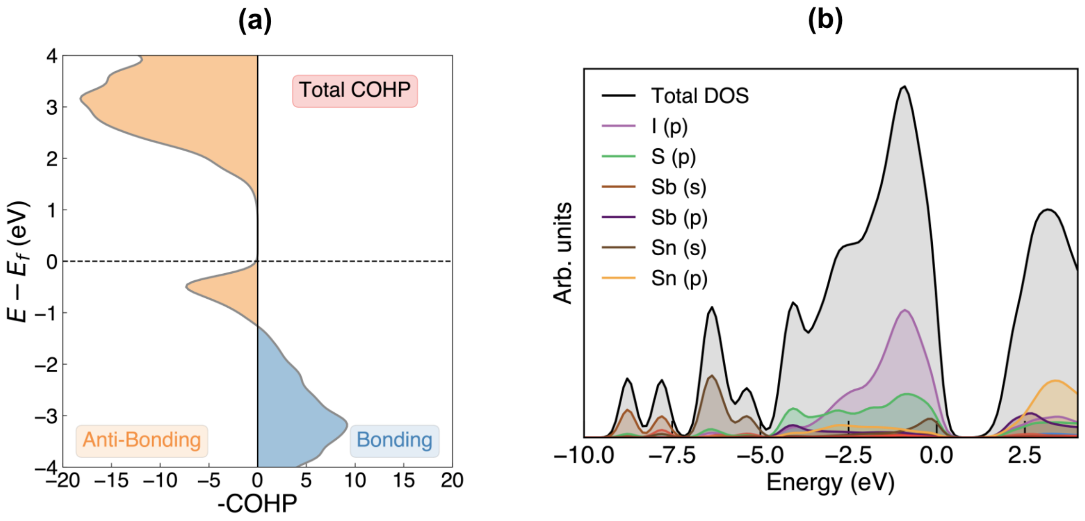

Figure S13: a) Cumulative Crystal Orbital Hamiltonian Population (COHP) analysis, which decomposes the density of states into bonding and anti-bonding orbital interactions.[S6,S13,S14] Negative COHP values (blue) indicate bonding-type interactions and positive values (orange) indicate anti-bonding interactions. b) Orbital-decomposed density of states calculated using the optB86b-vdW functional.

Shift Current Calculation

Shift current was calculated using the Wannier-interpolation scheme suggested by Ibañez-Azpiroz et al.[S15] From PBE functional DFT calculations, 144 maximally localised Wannier functions were disentangled from 480 bands with the maximum frozen window at least 10 eV above the Fermi energy using the `Wannier90` code.[S16] The interpolated band structures agreed well with the ones calculated directly from DFT calculation suggesting a high quality of Wannierisation. The shift current was calculated with frequency sampling of 0.005 eV and infinitesimal η was set to 0.04 eV.[S15] We validated the numerical convergence of the shift current by checking that the shift current vanishes for symmetry prohibited tensor elements and for centrosymmetric structures.

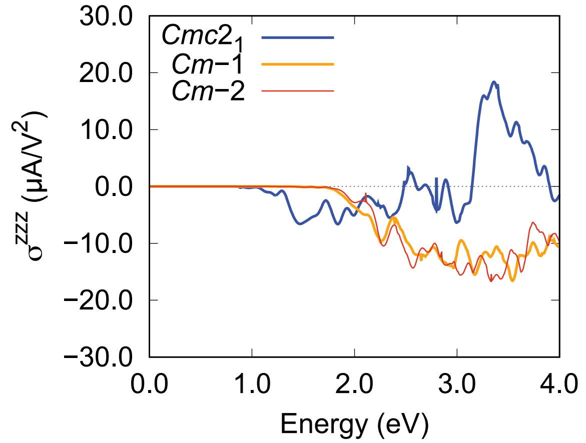

Figure S14: The zzz component of the shift current calculated for the $Cmc2_1$ and the two Cm structures.

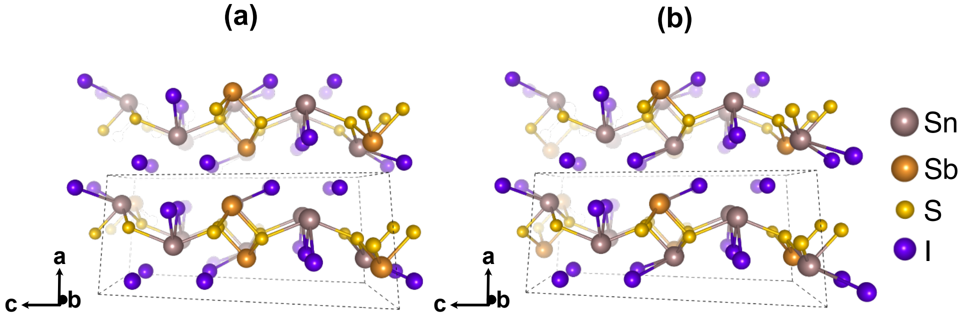

Figure S15: The two Cm phases within 2.0 meV/atom of the 0K ground state crystal structure. The atom colours are as follows: Sn = beige, Sb = orange, S = purple, I = purple. All figures generated using `VESTA`.[S3]

References

[S1] Ibanez, A.; Jumas, J. C.; Olivier-Fourcade, J.; Philippot, E. Mise en évidence d'un désordre statistique dans les structures chalcogénoiodures d'étain et d'antimoine. *Journal of Solid State Chemistry* **1984**, *55*, 83–91.

[S2] Doussier, C.; Moëlo, Y.; Léone, P.; Meerschaut, A.; Evain, M. Crystal Structure of $Pb_2SbS_2I_3$, and Re-Examination of the Crystal Chemistry within the Group of (Pb/Sn/Sb) Chalcogeno-Iodides. *Solid State Sciences* **2007**, *9*, 792–803.

[S3] Momma, K.; Izumi, F. VESTA: A Three-Dimensional Visualization System for Electronic and Structural Analysis. *Journal of Applied Crystallography* **2008**, *41*, 653–658.

[S4] Olivier-Fourcade, J.; Jumas, J. C.; Maurin, M.; Philippot, E. Mise en Évidence d'un Nouveau Sulfoiodure d'Étain et d'Antimoine $Sn_2SbS_2I_3$: Étude Structurale. *Zeitschrift für anorganische und allgemeine Chemie* **1980**, *468*, 91–98.

[S5] Kavanagh, S. R.; Savory, C. N.; Scanlon, D. O.; Walsh, A. Hidden Spontaneous Polarisation in the Chalcohalide Photovoltaic Absorber $Sn_2SbS_2I_3$. *Mater. Horiz.* **2021**, *8*, 2709–2716.

[S6] Nelson, R.; Ertural, C.; George, J.; Deringer, V. L.; Hautier, G.; Dronskowski, R. LOBSTER: Local Orbital Projections, Atomic Charges, and Chemical-Bonding Analysis from Projector-Augmented-Wave-Based Density-Functional Theory. *Journal of Computational Chemistry* **2020**, *41*, 1931–1940.

[S7] Müller, P. C.; Ertural, C.; Hempelmann, J.; Dronskowski, R. Crystal Orbital Bond Index: Covalent Bond Orders in Solids. *J. Phys. Chem. C* **2021**, *125*, 7959–7970.

[S8] Ganose, A. M.; Jackson, A. J.; Scanlon, D. O. Sumo: Command-line Tools for Plotting and Analysis of Periodic Ab Initio Calculations. *Journal of Open Source Software* **2018**, *3*, 717.

[S9] Klimeš, J.; Bowler, D. R.; Michaelides, A. Van Der Waals Density Functionals Applied to Solids. *Phys. Rev. B* **2011**, *83*, 195131.

[S10] Das, P.; Bazhirov, T. Electronic Properties of Binary Compounds with High Fidelity and High Throughput. *J. Phys.: Conf. Ser.* **2019**, *1290*, 012011.

[S11] Godby, R. W.; Schlüter, M.; Sham, L. J. Self-Energy Operators and Exchange-Correlation Potentials in Semiconductors. *Phys. Rev. B* **1988**, *37*, 10159–10175.

[S12] Zhu, B.; Kavanagh, S. R. Easyunfold. `https://github.com/SMTG-UCL/easyunfold`.

[S13] Deringer, V. L.; Tchougréeff, A. L.; Dronskowski, R. Crystal Orbital Hamilton Population (COHP) Analysis As Projected from Plane-Wave Basis Sets. *The Journal of Physical Chemistry A* **2011**, *115*, 5461–5466.

[S14] Dronskowski, R.; Bloechl, P. E. Crystal Orbital Hamilton Populations (COHP): Energy-Resolved Visualization of Chemical Bonding in Solids Based on Density-Functional Calculations. *The Journal of Physical Chemistry* **1993**, *97*, 8617–8624.

[S15] Ibañez-Azpiroz, J.; Tsirkin, S. S.; Souza, I. Ab Initio Calculation of the Shift Photocurrent by Wannier Interpolation. *Phys. Rev. B* **2018**, *97*, 245143.

[S16] Pizzi, G. et al. Wannier90 as a Community Code: New Features and Applications. *J. Phys.: Condens. Matter* **2020**, *32*, 165902.
